# Therapeutic potential of melatonin-pretreated human dental pulp stem cells (hDPSCs) in an animal model of spinal cord injury

**DOI:** 10.1038/s41598-024-78077-z

**Published:** 2024-11-15

**Authors:** Arvin Naeimi, Seyedeh Fatemeh Mousavi, Naser Amini, Mandana Golipoor, Hatef Ghasemi Hamidabadi

**Affiliations:** 1 Student Research Committee, School of Medicine, Guilan University of Medical Sciences, Rasht, Iran; 2 Cellular and Molecular Research Center, Iran University of Medical Sciences, Tehran, Iran; 3 Neuroscience Research Center, School of Medicine, Guilan University of Medical Sciences, Rasht, Iran; 4 Department of Anatomy & Cell Biology, Faculty of Medicine, Mazandaran University of Medical Sciences, Sari, Iran

**Keywords:** Melatonin, DPSCs, hDPSCs, Human dental pulp stem cells, Spinal cord injury, SCI, Stem cells, Neurology, Nanoscience and technology

## Abstract

Dental pulp stem cells (DPSCs) show potential for treating neurodegenerative and traumatic diseases due to their neural crest origin. Melatonin (MT), an endogenous neurohormone with well-documented anti-inflammatory and antioxidant properties, has shown promising results with MSCs in terms of engraftment, proliferation, and neuronal differentiation in animal SCI models. However, the effects of melatonin preconditioning on human dental pulp stem cells (hDPSCs) for SCI treatment remain unclear. This study investigates the impact of melatonin preconditioning on hDPSCs engraftment, neural differentiation, and neurological function in rats with SCI. Forty-two male Sprague–Dawley rats were divided into six groups: Control, Sham, Model, Vehicle, Lesion Treatment A (SCI + hDPSCs), and Lesion Treatment B (SCI + MT-hDPSCs). After obtaining hDPSCs, stem cells were evaluated using flow cytometry. Cell viability was assessed using the MTT assay. SCI was induced in the Model, Vehicle, Lesion Treatment A, and Lesion Treatment B groups. The Lesion Treatment A and B groups received hDPSCs and hDPSCs pretreated with melatonin, respectively, 1 week after SCI, while the Vehicle group received only an intravenous injection of DMEM to simulate treatment. The other groups were used for behavioral testing. Immunohistochemistry (IHC) was employed to assess hDPSCs engraftment and differentiation at the SCI site. Motor function across the six groups was evaluated using the Basso, Beattie, and Bresnahan (BBB) score. Histological studies and cell counts confirmed hDPSCs implantation at the injury site, with a significantly higher presence in the MT-hDPSCs compared to hDPSCs (*p* < 0.01). IHC revealed that hDPSCs and MT-hDPSCs differentiated into neurons and astrocytes, with greater differentiation observed in the MT-hDPSCs compared to the hDPSCs (*p* < 0.01 and *p* < 0.05, respectively). Functional improvement was noted in both SCI + hDPSCs and SCI + MT-hDPSCs groups compared to SCI and Vehicle groups from Week 4 onward (*p* < 0.001). Significant differences were also observed between the SCI + hDPSCs and SCI + MT-hDPSCs groups starting from Week 7 (*p* < 0.01). Preconditioning hDPSCs with melatonin enhances engraftment, neuronal differentiation, and greater performance improvement compared to hDPSCs alone in the SCI animal model.

## Introduction

Spinal cord injury (SCI) is a serious neurological disorder leading to permanent sensory and motor dysfunctions due to loss of neurons, glia and incomplete axonal regeneration after injury^1,2^. This devastating condition exerts an impact on the patient’s mental, physiological, and social well-being state^2^. Since there is no effective treatment for SCI, patients have to bear the significant financial burden of their healthcare and rehabilitation treatment. The incidence of SCI is growing worldwide, estimated at 10.4 to 83 cases per million annually, which also ranges from 20.7 to 83.0 in North America^3^. The development of advantageous treatments for SCI has been challenging due to its complicated pathophysiology. The pathophysiology of SCI is a biphasic event that is characterized by a primary injury followed by a secondary phase of injury^4^. During the acute phase, a primary focal mechanical insult disrupts tissue homeostasis, which triggers a secondary injury process. Then, the second phase activates resident microglia and infiltrates blood-derived macrophages to commence severe inflammation by releasing high levels of multiple neurotoxic factors, inducing necrotic and apoptotic death in neurons, oligodendrocytes, and astrocytes. This reaction spreads beyond the primary injury site and leads to irreversible demyelination and axonal damage^5^.

Despite existing challenges, there is growing interest in finding novel therapeutic strategies for efficient SCI repair^6^. Lately, in addition to pharmacologic therapy and surgical intervention, diverse therapeutic options have been tested to cure SCI, including cell-based strategies, neuroprotective factors, and neuroregenerative agents^7,8^. Although there are currently few neuroprotective and regenerative therapies that directly exert beneficial effects, cell therapies with neuroprotective and neuroregenerative properties may illustrate a new horizon in the treatment of SCI^9^. However, finding an ideal stem cell type to treat nerve injuries is challenging due to variations in the physiological and biological functions of different types of stem cells, feasibility of access, risk of post-transplant rejection, and high costs^10^. Hence, several types of stem cells have been investigated as potential therapeutic candidates for SCI, including central nervous system stem cells, mesenchymal stem cells (MSC), embryonic stem cells (ESC), induced pluripotent stem cells, and fetal-derived neural stem cells^9,11–13^. However, the utility of induced pluripotent stem cells(iPSC) and embryonic stem cells in the clinic is currently limited due to ethical and safety concerns^14^. So far, MSCs have been the most commonly studied cell type in SCI, and studies have shown MSCs/progenitor cells obtained from mesodermal tissues as a potential source for neural regeneration^6,15^.

To date, numerous populations of mesenchymal stem cells have been discovered, isolated, and characterized in several dental tissues, including dental pulp stem cells (DPSCs)^16,17^. Since DPSCs originate from neural crests, they possess remarkably higher neural stem cell properties compared to bone marrow‑derived mesenchymal stem cells. Other characteristics that indicate DPSCs as a good candidate for cell therapy are their easy accessibility, their potential for autologous transplantation, and their ability to produce trophic factors, including glial cell‑derived neurotrophic factor (GDNF), brain‑derived neurotrophic factor (BDNF), and ciliary neurotrophic factor (CNTF) that promote neuronal survival^3^. Moreover, DPSCs have also been shown to have enhanced neurogenic, angiogenic, and regenerative potential. DPSCs present an engrossing and versatile stem cell source for tissue engineering^6^. Despite the fact that stem cell therapy has shown promising clinical outcomes, some barriers, including the rejection of cell engraftment in the target tissue, remain a major obstacle^18^. Hence, studies have demonstrated that 80% of cells die within 72 h after transplantation^8^. It has been widely acknowledged that this issue is partly owing to the oxidative stress induced by reactive oxygen species (ROS) at the site of injury, leading to inhibition of cell proliferation, adhesion, and migration at the graft site. In this regard, research has shown that antioxidant preconditioning raises engraftment efficacy and stimulates cell proliferation^19,20^.

Melatonin, an endogenous hormone produced by the pineal gland, is one of the preconditioning antioxidants proven to promote cell viability. This hormone is associated with angiogenesis and inflammatory pathways and, at the molecular level, acts as an antioxidant by removing ROS and reducing endoplasmic reticulum stress (ERS)^21,22^. In addition, preconditioning of adipose-derived stem cells (ADSCs) and bone marrow stem cells (BMSCs) with melatonin has been shown to enhance cell viability in various conditions, including liver fibrosis, ischemic kidney, and neural stem cells^8,22^. However, the role of melatonin preconditioning on human dental pulp stem cells (hDPSCs) for SCI treatment is still obscure. This study examines the effects of melatonin preconditioning on hDPSCs engraftment, neural differentiation, and neurological function in rats with SCI.

## Results

### hDPSCs isolation and culture

**Fig. 1:**
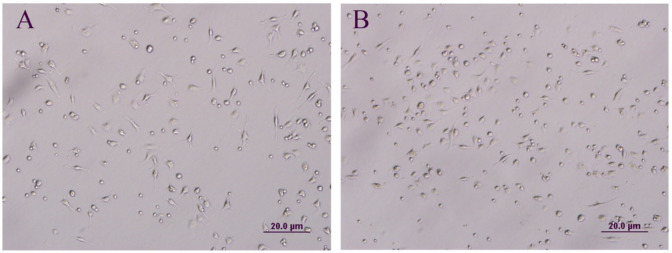
The morphology and confluency of hDPSCs and MT-hDPSCs. (**A**) Undifferentiated hDPSCs and (**B**) undifferentiated MT-hDPSCs display a flattened fibroblast-like morphology under phase-contrast microscopy. Within 4–5 days in culture medium, MT-hDPSCs (60–70%) reached higher confluency than hDPSCs (30–40%) (Magnification: ×20). *hDPSCs* human dental pulp stem cells, *MT* melatonin.

According to isolation and culture findings, MT-hDPSCs achieved higher confluency (60–70%) compared to hDPSCs (30–40%) within 4–5 days in the culture medium (Fig. 1).

### Flow cytometry analysis of hDPSCs

**Fig. 2:**
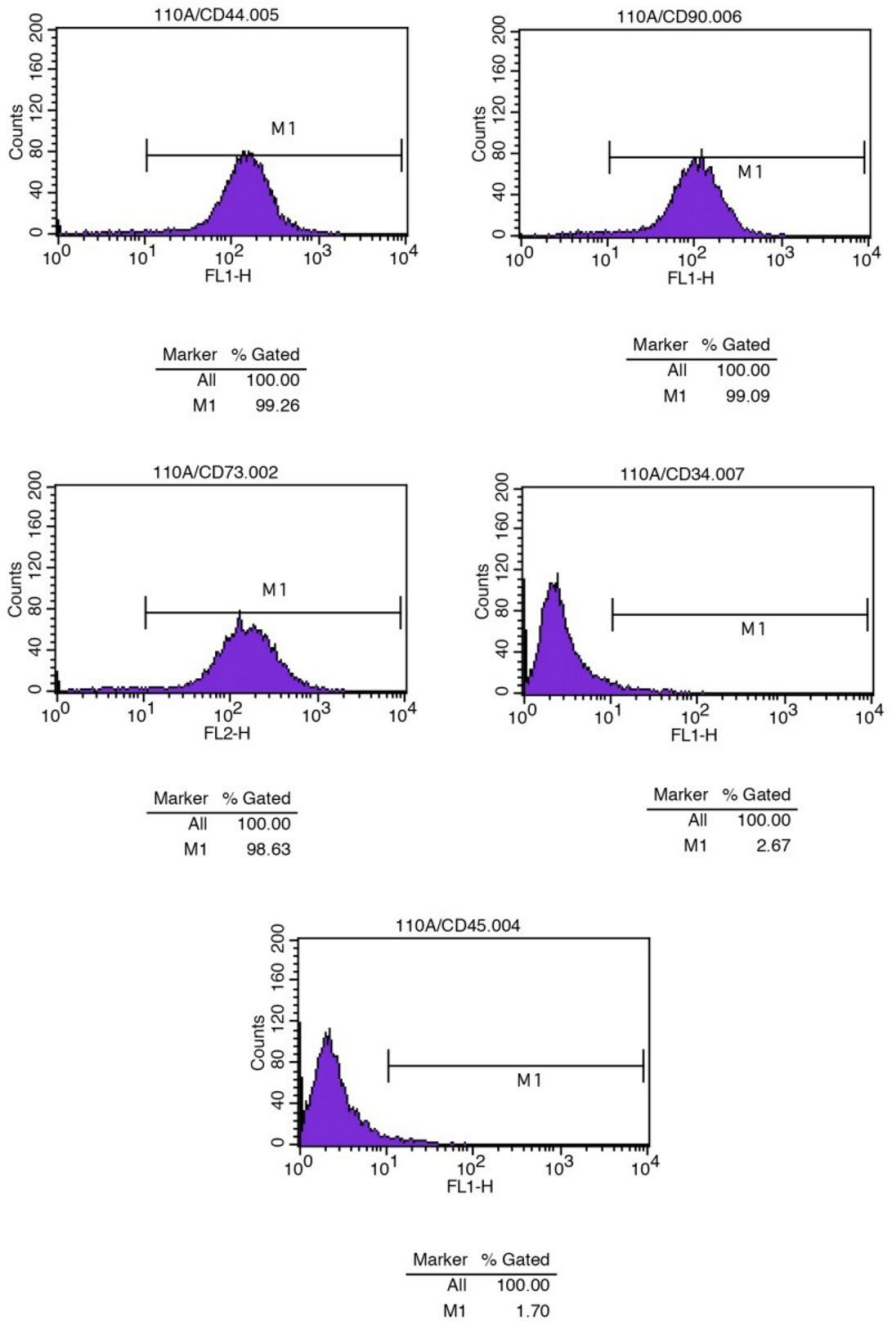
Isolation and identification of human dental pulp stem cells (hDPSCs). The expression of hDPSC surface markers was detected by flow cytometry. The cells were positive for CD44, CD90, and CD73 and negative for CD34 and CD45.

The flow cytometric analysis revealed positive expression of cell surface markers (CD44, CD90, and CD73) and negative expression of markers (CD45 and CD34), indicating the mesenchymal nature of hDPSCs (Fig. 2).

### Cell viability assay

**Fig. 3:**
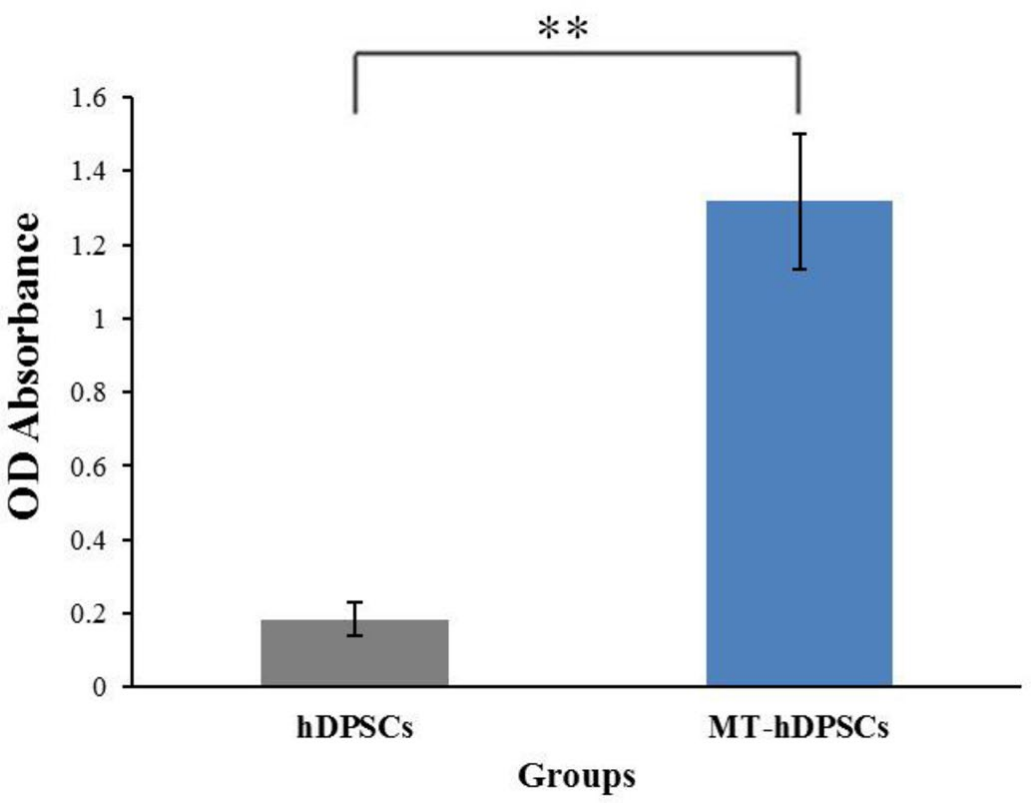
Cell viability assay. The results of the MTT assay showed a significantly higher optical density (OD) absorbance in the MT-hDPSCs group (1.31 ± 0.41) compared to the hDPSCs group (0.18 ± 0.10), indicating enhanced cell viability in MT-hDPSCs. This difference was statistically significant (*p* < 0.01).

The analysis of the MTT assay between the MT-hDPSCs and hDPSCs groups revealed that the optical density (OD) absorbance of the MT-hDPSCs group (1.31 ± 0.41) was significantly higher than that of the hDPSCs group (0.18 ± 0.10), indicating increased cell viability in MT-hDPSCs compared to hDPSCs (*p* < 0.01, Fig. 3).

### Confirmation model

**Fig. 4:**
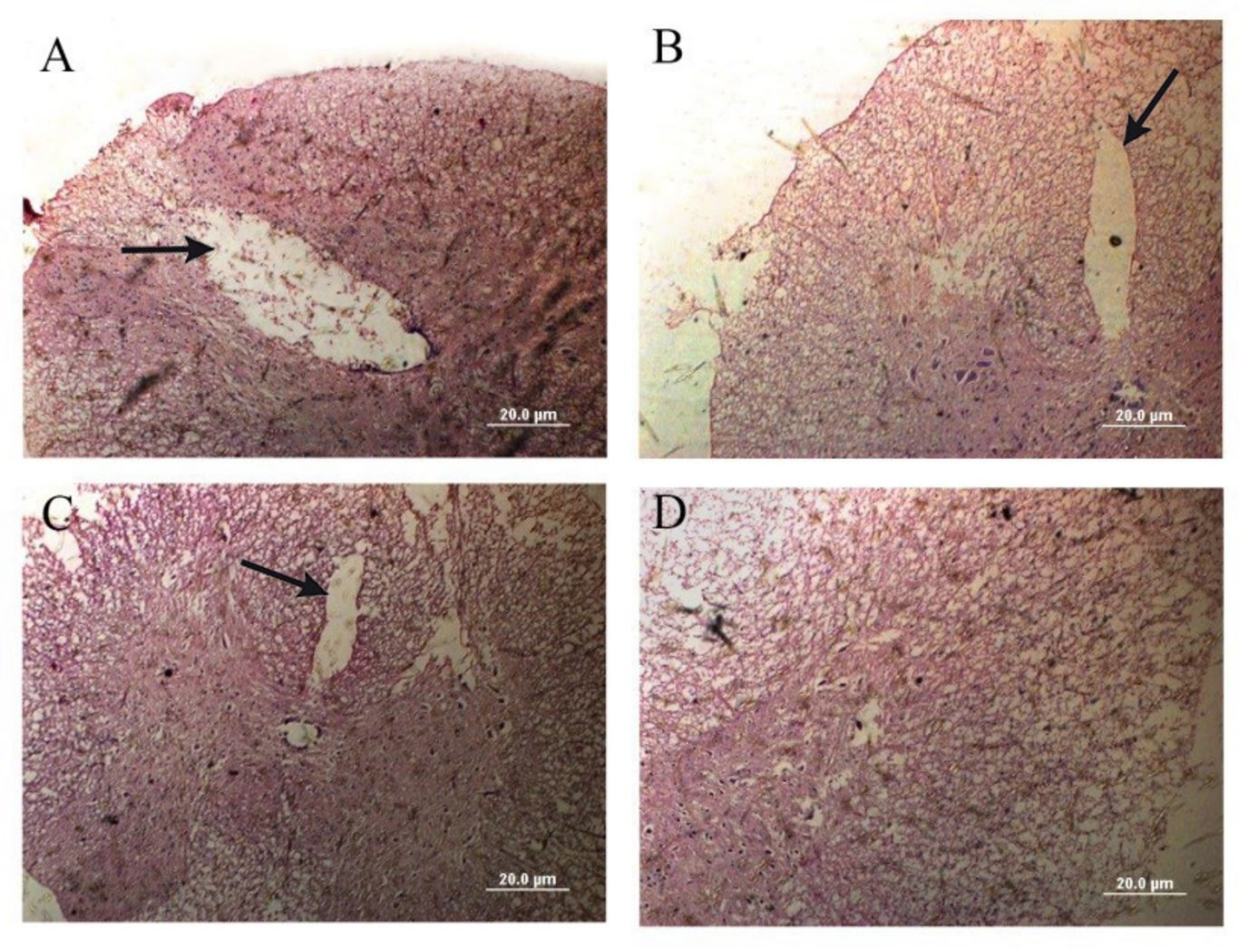
Spinal cord injury confirmation model. Hematoxylin and eosin (H&E) staining of spinal cord lesions demonstrated that the (**D**) Control group had normal spinal tissue. In contrast, the (**A**) Vehicle (SCI + DMEM), (**B**) SCI + hDPSCs, and (**C**) SCI + MT-hDPSCs groups displayed hollow cystic cavities at the injury site, confirming the presence of spinal injury (Magnification: ×20). *hDPSCs* human dental pulp stem cells, *MT* melatonin, *SCI* spinal cord injury.

H&E staining confirmed the presence of hollow cystic cavities at the SCI site in all groups, including Vehicle (SCI + DMEM), SCI + hDPSCs, and SCI + MT-hDPSCs, thus verifying the SCI model. Additionally, the therapeutic groups of SCI + hDPSCs and SCI + MT-hDPSCs showed a reduction in size and disappearance of cystic cavities (Fig. 4).

### The localization of hDPSCs and MT-hDPSCs at the SCI site

**Fig. 5:**
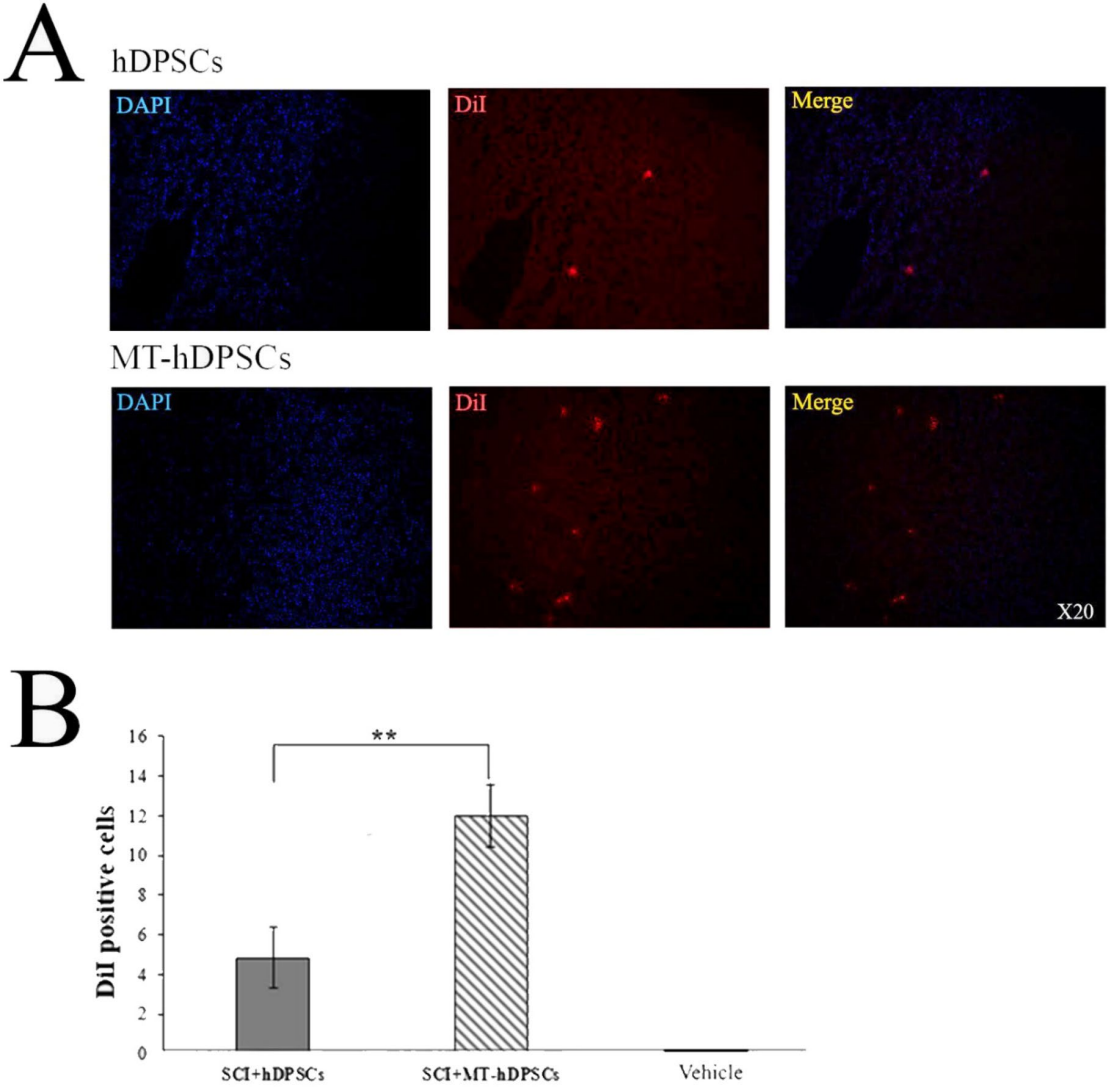
Evaluation of hDPSCs and MT-hDPSCs at the SCI site. (**A**) At the site of injury, hDPSCs and MT-hDPSCs were labeled with DiI, and their nuclei were labeled with DAPI. (**B**) A comparison of the number of DiI-positive cells among the Vehicle (SCI + DMEM), SCI + hDPSCs, and SCI + MT-hDPSCs groups showed that the number of MT-hDPSCs at the SCI site was significantly higher than that of hDPSCs (**p < 0.01). *hDPSCs* human dental pulp stem cells, *MT* melatonin, *SCI* spinal cord injury; magnification: ×20.

IHC analysis revealed the presence of hDPSCs and MT-hDPSCs at the SCI site, with an average of 4.83 ± 1.537 hDPSCs and 12 ± 1.592 MT-hDPSCs observed in the field of view at 20X magnification, suggesting a higher engraftment of MT-hDPSCs at the lesion site [t (10) = 3.239, *p* < 0.01] (Fig. 5).

### Differentiation of hDPSCs and MT-hDPSCs into neurons

**Fig. 6:**
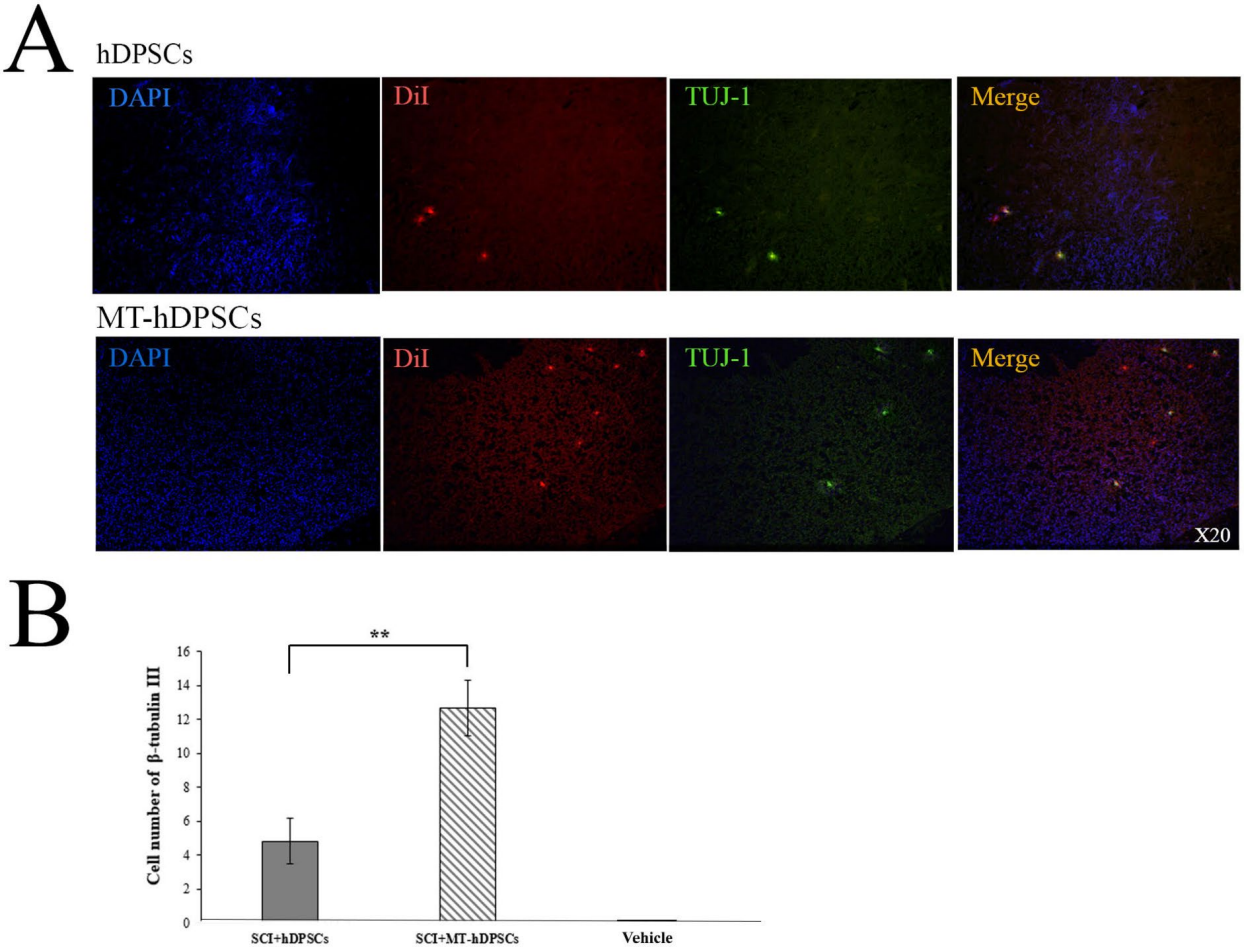
Differentiation of hDPSCs and MT-hDPSCs into neurons assessed by β-Tubulin III (TUJ-1) staining. (**A**) At the site of injury, the nuclei of hDPSCs and MT-hDPSCs were labeled with DAPI, the cells themselves were labeled with DiI, and their neuron differentiation was labeled with β-tubulin III. (**B**) A comparison of the number of β-tubulin III-positive cells among the Vehicle (SCI + DMEM), SCI + hDPSCs, and SCI + MT-hDPSCs groups demonstrated that MT-hDPSCs differentiated into neurons significantly more than hDPSCs at the SCI site (**p < 0.01). *hDPSCs* human dental pulp stem cells, *MT* melatonin, *SCI* spinal cord injury; magnification: ×20.

β-tubulin III (TUJ-1) staining was conducted in the Vehicle, SCI + MT-hDPSC, and SCI + hDPSC groups to assess the differentiation of hDPSCs and MT- hDPSCs into neurons. The results indicated that β-tubulin III (TUJ-1) expression was present in both experimental groups receiving hDPSCs and MT- hDPSCs. Furthermore, the number of β-tubulin III stained cells at the SCI site was significantly higher in the SCI + MT-hDPSCs group (12.67 ± 1.647) compared to the SCI + hDPSCs group (4.83 ± 1.352) [t(10) = 3.677, *p* < 0.01)] (Fig. 6).

### Differentiation of hDPSCs and MT-hDPSCs into astrocytes

**Fig. 7:**
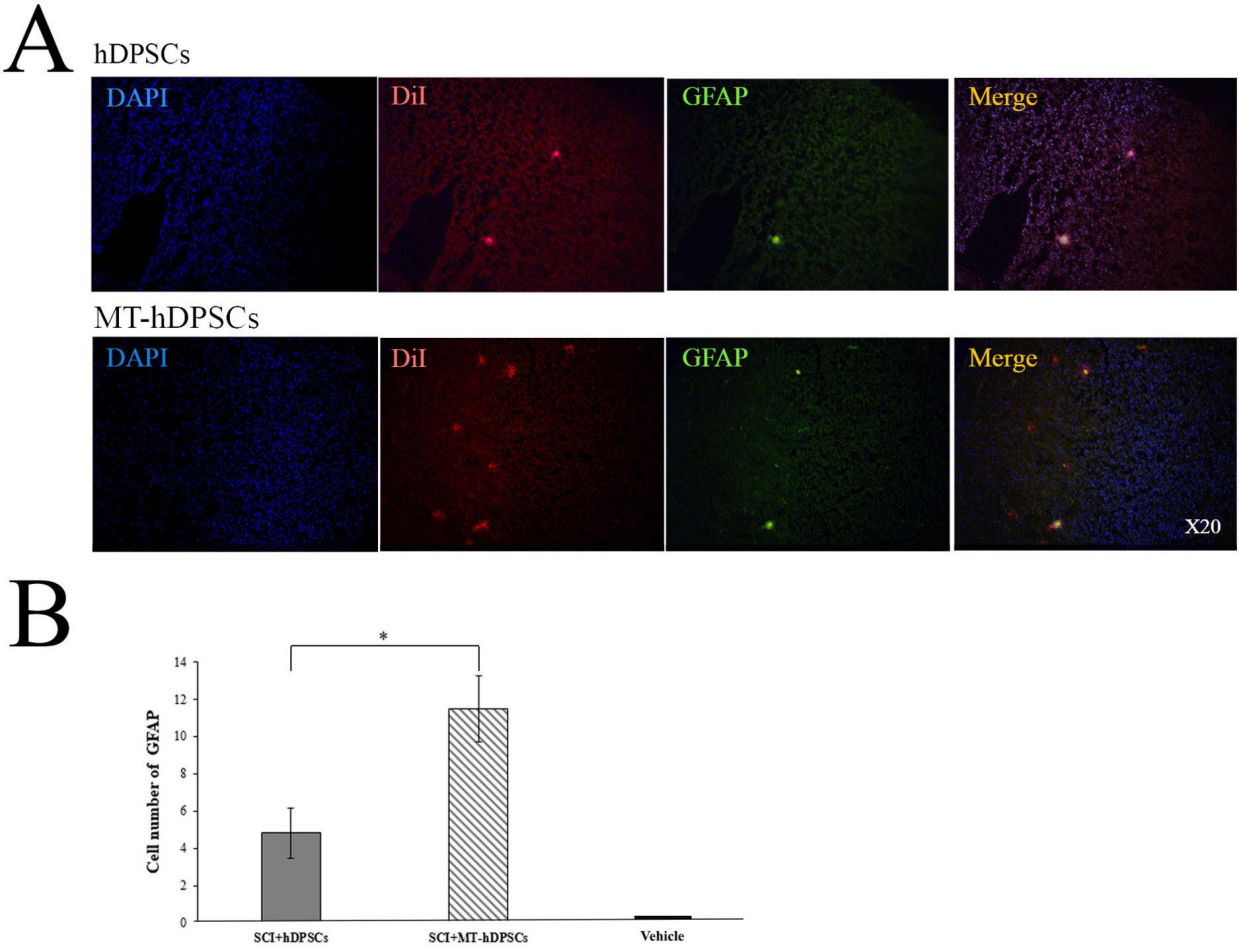
Differentiation of hDPSCs and MT-hDPSCs into astrocytes according to GFAP staining. (**A**) At the site of injury, the nuclei of hDPSCs and MT-hDPSCs were labeled with DAPI, the cells were labeled with DiI, and their differentiation into astrocytes was labeled with GFAP. (**B**) A comparison of the number of GFAP-positive cells among the Vehicle (SCI + DMEM), SCI + hDPSCs, and SCI + MT-hDPSCs groups revealed that MT-hDPSCs differentiated into astrocytes more than hDPSCs at the SCI site (*p < 0.05). *hDPSCs* human dental pulp stem cells, *MT* melatonin, *SCI* spinal cord injury; magnification: ×20.

GFAP staining was performed on the Vehicle, SCI + MT-hDPSC, and SCI + hDPSC groups to evaluate the differentiation of hDPSCs and MT- hDPSCs into astrocytes. GFAP expression was observed in both experimental groups. However, the number of GFAP-stained cells at the SCI site was significantly higher in the SCI + MT- hDPSC group (11.5 ± 1.76) compared to the SCI + hDPSC group (4.83 ± 1.35) [t(10) = 2.998, *p* < 0.05] (Fig. 7).

### BBB score evaluation

**Fig. 8:**
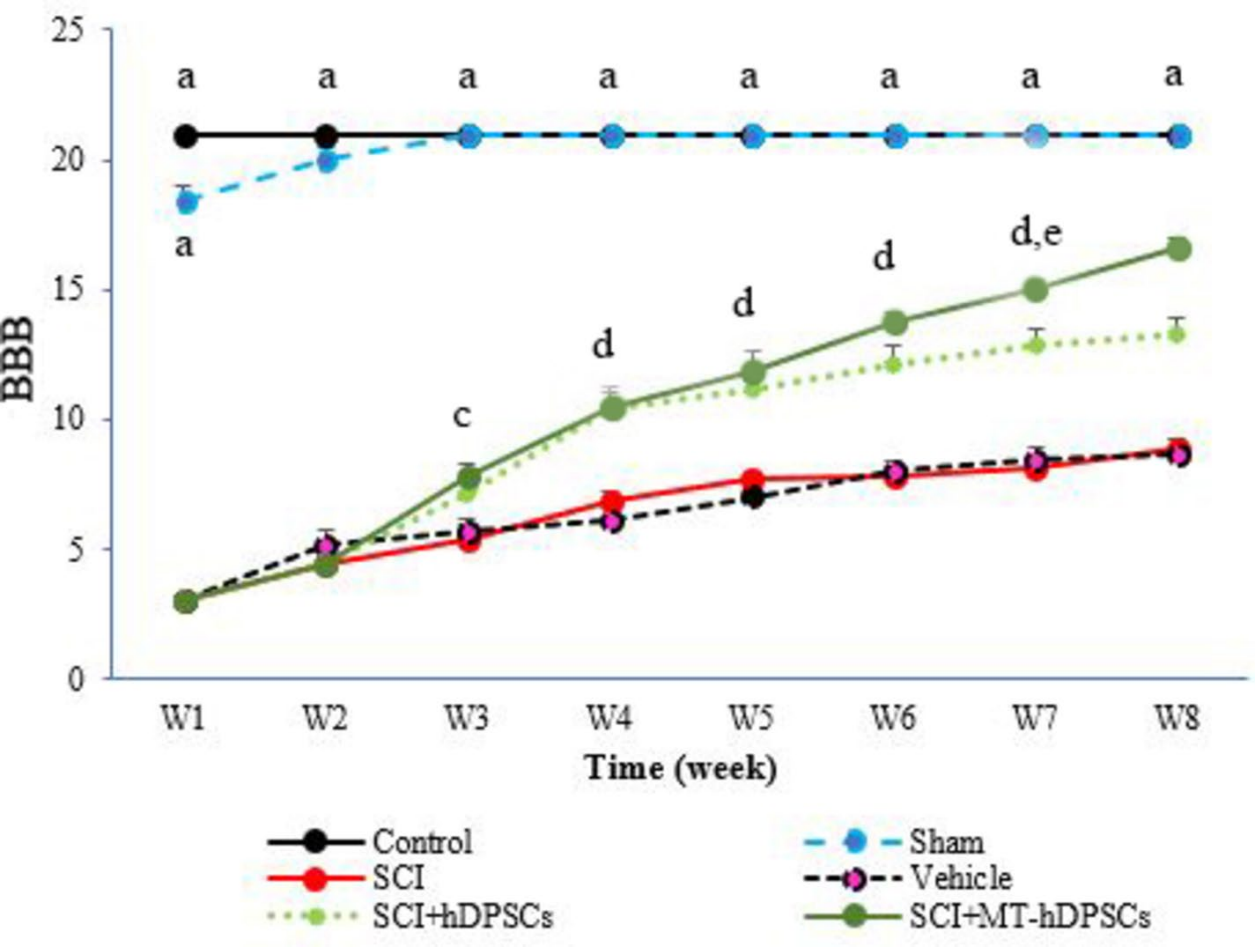
Assessment of Basso, Beattie, and Bresnahan (BBB) scores in the Control, Sham, Model (SCI), Vehicle (SCI + DMEM), SCI + hDPSCs, and SCI + MT-hDPSCs groups. During the first week after cell transplantation, significant differences were noted between the Control group and the other groups (Week 1: f(5, 41) = 1205.28, *p* < 0.001). By the fourth week, the BBB scores for the SCI + MT-hDPSCs and SCI + hDPSCs groups were significantly higher compared to the SCI and Vehicle groups (Week 4: f(5, 41) = 198.639, *p* < 0.001). Additionally, the BBB scores for the Control and Sham groups were significantly higher than those of the other groups. These significant differences persisted through the fifth week (Week 5: f(5, 41) = 151.80, *p* < 0.001), sixth week (Week 6: f(5, 41) = 204.731, *p* < 0.001), seventh week (Week 7: f(5, 41) = 191.104, *p* < 0.001), and eighth week (Week 8: f(5, 41) = 197.617, *p* < 0.001), with the SCI + MT-hDPSCs and SCI + hDPSCs groups showing significant improvements compared to the SCI and Vehicle groups. Significant differences were also observed between the SCI + hDPSCs and SCI + MT-hDPSCs groups throughout the testing period, starting from Week 7 (*p* < 0.01). a. Indicates *P* < 0.001 for the Control and Sham groups versus the SCI, Vehicle, SCI + ADSC, and SCI + MT-ADSC groups; d. Indicates *P* < 0.001 for the SCI + hDPSCs and SCI + MT-hDPSCs groups compared to the SCI group; c. Indicates *P* < 0.001 for the SCI + hDPSCs and SCI + MT-hDPSCs groups compared to the Control, Sham, and SCI groups; e. Indicates *P* < 0.01 for the SCI + MT-hDPSCs group versus the SCI + hDPSCs group. hDPSCs: human dental pulp stem cells; MT: melatonin; SCI: spinal cord injury.

In the first week after cell transplantation, motor test scores were low across all groups except the Control group, which showed significant differences compared to the other groups (Week 1: f (5,41) = 1205.28, *p* < 0.001). However, by the fourth week, the BBB scores for the SCI + MT-hDPSCs and SCI + hDPSCs groups were significantly higher compared to the SCI and Vehicle groups (Week 4: f (5,41) = 198.639, *p* < 0.001); Additionally, the BBB scores for the Control and Sham groups were significantly higher than those of the other groups. Significant differences persisted in the fifth week (Week 5: f (5,41) = 151.80, *p* < 0.001), sixth week (Week 6: f (5,41) = 204.731, *p* < 0.001), seventh week (Week 7: f (5,41) = 191.104, *p* < 0.001), and eighth week (Week 8: f (5,41) = 197.617, *p* < 0.001), with the SCI + MT-hDPSCs and SCI + hDPSCs groups showing significant differences from the SCI and Vehicle groups. Significant differences were also observed between the SCI + hDPSCs and SCI + MT-hDPSCs groups throughout the testing period, starting from Week 7 (*p* < 0.01; Fig. 8).

## Discussion

Our study indicated that pretreatment of human dental pulp stem cells (hDPSCs) with melatonin (MT) enhances their viability and potentially promotes their proliferation. As a result, the MT-hDPSCs achieved a higher cell density within 4–5 days. Furthermore, the confluency level of melatonin-pretreated hDPSCs (MT-hDPSCs) was notably higher compared to untreated hDPSCs, reaching 60–70% before transplantation. These findings are consistent with our previous research, which demonstrated the positive impact of melatonin on ADMSCs viability and proliferation, as well as a higher cell density in MT- ADMSCs compared to untreated ADMSCs within the same time frame^23^. Various studies have suggested that melatonin may enhance the rate of cell proliferation in certain cell types^24–28^. In line with our findings, numerous authors have reported that melatonin promotes the viability^29^ and proliferation^24,30,31^ of DPSCs at certain concentrations. However, some studies have found that melatonin does not affect the viability of hDPSCs^30^ and may even reduce DPSCs proliferation^29^. These inconsistencies may be due to variations in melatonin concentrations and conditioning periods used in different studies. Some research has explored the dose-dependent effects of melatonin on the viability^31,32^, proliferation, and differentiation of DPSCs^31^, suggesting that concentrations of 1 µM and 25 µM are ideal for pretreatment, as they are well-tolerated without inducing toxic effects. Additionally, other studies have shown that treatment with 0.1 µM and 10 µM melatonin for four days promotes DPSCs viability^31^. Authors have also demonstrated that hDPSCs treated with intermediate concentrations of melatonin (10–300 µM) displayed significantly higher proliferation than untreated counterparts^30^. In our study, we selected 5 µM melatonin for pretreatment based on previous experiments^33^, finding that this concentration enhances cell viability and potentially promotes proliferation within 4–5 days. Overall, melatonin inhibits the production of inflammatory cytokines, encourages the proliferation of MSCs, and protects these cells from apoptosis, according to in vitro and in vivo research. The anti-apoptotic and protective actions of melatonin involve regulating the genes that produce ROS and antioxidants^24^. However, although different types of MSCs share common phenotypic characteristics, they may not respond similarly to the same stimuli. In other words, melatonin’s effects on MSC proliferation depend on the MSC type and the melatonin concentration^24,28^. In our study, the number of engrafted hDPSCs pretreated with melatonin was significantly higher at the SCI site compared to untreated hDPSCs. This increase could be attributed to enhanced cell migration, increased cell proliferation, or reduced apoptosis^33^. The role of melatonin in cell migration has been demonstrated in other studies as well. For instance, García-Bernal et al. have shown that melatonin (10–300 µmol/L) positively affects hDPSC migration^30^. Furthermore, others reported that melatonin stimulates the motility of umbilical cord MSCs through melatonin receptor 2 signaling, which involves the downstream phosphorylation of focal adhesion kinase and paxillin, increased levels of active Cdc42 and Arp2/3, and the stimulation of cytoskeletal proteins such as profilin 1, cofilin 1, and F-actin^23^. Additionally, melatonin has been shown to exert various receptor-mediated effects on MSCs, including enhanced survival, motility, engraftment, and cell differentiation. These effects appear to be linked to interactions between receptors and matrix enzymes, resulting in improved homing of MSCs following the pre-administration of a combination of melatonin and MSCs^23,34,35^. Moreover, melatonin is known to reduce oxidative stress^36^, creating a more favorable microenvironment that promotes stem cell survival and recruitment to the injury site^36^. These findings suggest that melatonin’s effects on hDPSC migration and proliferation may be advantageous for tissue restoration by regulating both MSC migration and self-renewal in vivo^30^. Moreover, the combination of increased migratory capacity and improved survival in a supportive environment may explain why melatonin-pretreated hDPSCs are found in significantly greater numbers at the SCI site compared to their untreated counterparts, ultimately leading to enhanced therapeutic outcomes in tissue repair and regeneration.

The current study revealed that hDPSCs and MT-hDPSCs differentiate into neurons and astrocytes, with greater differentiation observed in the MT-hDPSCs compared to the hDPSCs. To date, several studies have demonstrated the differentiation capability of human dental pulp stem cells into mature neurons^6,37^, oligedencrocyte^37^, neuron-like cells (characterized with neuron-specific nuclear protein (NeuN), tyrosine hydroxylase neuronal markers and by morphological features)^32^, cholinergic neurons (via nerve growth factor)^38^, motor neuron-like cells^39^, astrocytes and Schwann cells^40^. In this regard, numerous studies have utilized DPSCs in animal SCI models^6,32,37,40–43^. Consistent with our findings, some studies demonstrated that hDPSCs were able to express some glial markers, such as GFAP, and differentiate from astrocytes in a mouse SCI model^40^. Moreover, some others demonstrated that IV-administered hDPSCs migrate to the lesion boundary area in the ischemic rat brain and differentiate into astrocytes and neurons^44^. Besides, authors have shown that pretreatment and post-treatment with hDPSCs or CM-hDPSCs provide superior cytoprotective effects on astrocytes in an in vitro ischemic model compared to hMSCs, likely due to reduced reactive gliosis and the suppression of free radicals and the proinflammatory cytokine IL-1β expression^45^. Despite this, some studies indicate that while DPSCs can differentiate into mature neurons and oligodendrocytes, they do not differentiate into astrocytes^46^. Overall, transplanted DPSCs have been shown to offer distinct therapeutic advantages for the treatment of SCI, including (1) suppression of the early inflammatory response; (2) inhibition of SCI-induced apoptosis in neurons, astrocytes, and oligodendrocytes, thereby promoting the preservation of neural fibers and myelin sheaths; (3) regeneration of transected axons through the direct inhibition of multiple axon growth inhibitors signals (such as chondroitin sulfate proteoglycans and myelin-associated glycoprotein) via paracrine mechanisms; and (4) cell replacement in the damaged spinal cord through the differentiation capacity of pulp stem cells into oligodendrocytes, neurons, and astrocytes^5^. Besides, DPSCs have been found to exhibit a higher level of hepatocyte growth factor (HGF) expression, further supporting their potential use in spinal cord repair^47^. HGF has previously been reported to exert a neurotrophic effect on CNS neurons, including spinal motor neurons, and also acts as an axon chemoattractant^48,49^. More recently, Kitamura et al. reported that HGF enhances endogenous repair and promotes functional recovery following SCI^50^. Animals treated with HGF-expressing MSCs showed increased axonal growth beyond the glial scar and improved forepaw function after SCI^5^. Additionally, HGF was shown to inhibit glial scar formation by reducing astrocytic activation. The inadequate blood supply following SCI is a significant barrier to regeneration, underscoring the importance of angiogenesis in the endogenous regenerative response after injury. FGF2 and EGF have already been implicated in angiogenesis. VEGF, a key pro-angiogenic regulator, has also been shown to be expressed by dental stem cells (DSCs), and its expression, along with FGF, was upregulated following exposure to interferon-gamma (IFN-γ). VEGF has further been implicated in neurogenesis and has been shown to exhibit both neurotrophic and neuroprotective properties^5^.

Despite numerous promising results with DPSCs in SCI treatment, significant challenges remain in using DMSCs for SCI regeneration, including the low rate of cell engraftment and survival after transplantation^46^. In this regard, we found that preconditioning hDPSCs with melatonin enhanced cell engraftment and survival and promoted their differentiation into neurons and astrocytes, compared to untreated hDPSCs. These results align with our previous findings on the impact of melatonin on ADSCs engraftment and neuronal differentiation^33^. Besides, in the present study, functional improvements were observed in both the SCI + hDPSCs and SCI + MT-hDPSCs groups compared to the SCI and Vehicle groups starting from Week 4. Consistent with these findings, other studies have shown that hDPSCs facilitate locomotor function recovery in SCI models^37,41^. Moreover, we found significant differences between the SCI + hDPSCs and SCI + MT-hDPSCs groups from Week 7 onward. Overall, melatonin has garnered attention for its ability to enhance cell activities and preserve the functional integrity of MSCs by regulating reactive oxygen species generation and anti-inflammatory responses^24^. However, while melatonin’s effects have been explored in various MSC types, its role in melatonin pretreatment of DPSCs within SCI models and the underlying mechanisms are still limited. Melatonin has been shown to induce neuronal differentiation by increasing YAPY357 phosphorylation and inhibiting YAP activity, suggesting an active Hippo signaling pathway^51^. Additionally, some studies indicate that melatonin enhances the immunoregulatory and anti-inflammatory properties of hDPSCs, with increased soluble TGF-β secretion playing a key role in the observed immunomodulatory effects. Furthermore, melatonin promotes the expression of CD146 in DPSCs, which is associated with enhanced self-replication and immunomodulation^24^. Despite these insights, further research is needed to fully elucidate the mechanisms underlying the effects of melatonin-pretreated hDPSCs in SCI models.

### Limitations

While this study highlights the positive effects of melatonin-pretreated hDPSCs on engraftment, neural differentiation, and functional recovery in a spinal cord injury model, there are a few important limitations to consider. One key limitation is that we did not conduct in-depth molecular assessments, such as analyzing the expression of neurotrophic factors (NTFs), which are known to support nerve protection and repair. Moreover, although we used immunohistochemistry (IHC) to examine cell engraftment and differentiation, we did not specifically measure NTF expression at the injury site. Future studies should focus on incorporating gene expression analysis and IHC for important NTFs, such as brain-derived neurotrophic factor (BDNF), glial cell line-derived neurotrophic factor (GDNF), and nerve growth factor (NGF), to enhance our understanding of how melatonin-pretreated hDPSCs contribute to neural recovery.

## Conclusion

Preconditioning hDPSCs with melatonin enhances engraftment, neuronal differentiation, and overall performance improvement compared to hDPSCs alone in an SCI animal model.

## Methods

### Animals

Forty-two eight-nine-week-old male Sprague-Dawley rats, each weighing about 250–300 g, were provided from the Animal Care Center of Guilan University of Medical Sciences. The rats were housed in an animal room with a constant temperature (22–25 °C) and humidity level (50-60%) with a 12/12 light/dark cycle. The rats had ad libitum access to a standard diet and water. The experimental protocols were approved by the Ethical Committee of the Guilan University of Medical Sciences (Helsinki Declaration) and were conducted in accordance with animal ethics guidelines of the national health and research institutes. The Rats were divided randomly into six groups (*n* = 7) as outlined below:

1. Control: This group of rats did not receive any treatment or injury.
2. Sham (Laminectomy): This group underwent surgical stress.
3. Model (SCI): This group experienced lesions without any therapeutic intervention.
4. Vehicle (SCI + DMEM): DMEM was administered intravenously to this group one week after SCI.
5. Lesion treatment A (SCI + hDPSCs): One week after SCI, 1 × 10^6^ cells/200 µl of hDPSCs was administered intravenously to this group.
6. Lesion treatment B (SCI + MT-hDPSCs): One week after SCI, 1 × 10^6^ cells/200 µl of hDPSCs pretreated with melatonin was transplanted intravenously to this group.

### Isolation and culture of hDPSCs

The isolation of hDPSCs was accomplished according to the protocol modified by Gronthos et al.^16^. Briefly, discarded normal human-impacted third molars were collected from 18 to 25 years old patients after obtaining informed consent from the participants. The extracted teeth were rinsed with phosphate-buffered saline (PBS, Gibco) containing 1% (v/v) penicillin-streptomycin (Invitrogen, USA). Dental pulp tissues were carefully removed from the dental cavities and minced into small pieces using sterile surgical scissors. The pulp tissues were then enzymatically digested with 3 mg/mL collagenase type I (Roche Diagnostics) and 4 mg/mL dispase (Sigma-Aldrich) for 1 h at 37 °C with gentle agitation. To eliminate any cellular debris, the mixture was filtered through a 70-µm-pore-size nylon mesh. The cells were centrifuged at 400 g for 5 min and cultured in DMEM/F12 (1:1) (GIBCO-BRL) supplemented with 10% fetal bovine serum (FBS; Invitrogen), 2 mM L-glutamine (Sigma-Aldrich), and 1% (v/v) penicillin-streptomycin. The cells were incubated at 37 °C with 5% CO2 in a humidified chamber. Upon reaching 80% confluency, the cells were trypsinized and passaged at a 1:3 ratio^39^. The cultures were subsequently expanded through two additional successive subcultures, with the medium being replaced every two days. The cells were monitored daily under an inverted microscope.

### Flow cytometry analysis of adult human dental pulp stem cells

To confirm the isolated cells as hDPSCs, the expression levels of positive markers (CD90, CD73, and CD44) and negative markers (CD45 and CD34) on the cell surface were assessed. Briefly, hDPSCs at the third passage were harvested using 0.25% trypsin and 0.02% EDTA, followed by washing with 0.01 M PBS. Subsequently, suspensions containing 50 ml at a cell concentration of 10^6^/ml were used as the test samples. These samples were then incubated for 20 min with anti-human CD90, CD73, CD45, CD44, and CD34 antibodies (BD Bioscience, USA). After being rinsed twice in PBS, the cells were analyzed using an NxT Flow Cytometer (AttuneTM; Thermo Fisher Scientific, USA).

### Melatonin pretreatment

To pretreat with melatonin, hDPSCs were subjected to incubation with 5 µM melatonin (M5250-1G, Sigma, USA) for 24 h following the third passage^33,52,53^. Subsequently, all preconditioned cells underwent three washes with PBS to eliminate any remaining melatonin prior to subsequent analysis.

### Cell labeling

The hDPSCs were suspended in a complete medium at a concentration of 1 × 10^6^ cells/mL and labeled with 1 µM CellTracker™ Cm-dil (Qcbio Science & Technologies Co., Ltd., Shanghai, China). The labeling process involved incubation at 37 °C for 5 min, followed by a 10-minute incubation at 4 °C. Afterward, the cells were washed three times with PBS and then harvested for cell transplantation.

### Cell viability assay

The MTT assay, using 3-(4, 5-Dimethylthiazol-2-yl)-2, 5-diphenyltetrazolium bromide (MTT, Sigma, USA), measures mitochondrial metabolic activity to indicate the number of viable cells in culture. In brief, cells were seeded into a 96-well plate at a density of 1.0 × 10⁶ cells per well. After washing with PBS, 100 µl of culture medium containing 50 µl of MTT reagent was added to each well. The cells were then incubated at 37 °C with 5% CO₂ for 1 h. After incubation, 200 µl of dimethyl sulfoxide (DMSO) was added to dissolve the formazan crystals, and absorbance was measured at 630 nm using an ELISA reader.

### The spinal cord injury model

We employed Lee et al.‘s clip compression method for inducing SCI^54^. Following anesthesia with ketamine (60 mg/kg, Dr. Mojallali Co., Tehran, Iran) and xylazine (20 mg/kg, Dr. Mojallali Co., Tehran, Iran), clip compression injury was induced at the 9-10th thoracic segments by surgically removing their dorsal processes under sterile conditions. The wound was then closed with sutures. Postoperatively, rats received care, including daily subcutaneous administration of cefazolin (50 mg/kg, Sobhan Darou Pharmaceutical Company, Iran) for 5 days and twice-daily abdominal compression to empty their urinary bladders. Three days after spinal cord injury, animals were examined, and those with a Basso, Beattie, and Bresnahan (BBB) score higher than three were excluded. Additionally, five cross-sections of micro-straws were made from the lesion site to confirm the model’s validity^54^.

### BBB score

Following cell transplantation, we utilized the BBB score on a weekly basis to assess and monitor the neurological function of the animals^55^ for a duration of up to 8 weeks. The BBB scale, established by the Multicenter Animal Spinal Cord Injury Study (MASCIS), serves as the primary metric for evaluating motor recovery in rats with spinal cord injuries^56^. This scale assigns scores ranging from 0 (indicating no observable hind limb movement) to 21 (representing normal locomotion). Rats with scores between 0 and 7 (indicating the early stage of recovery) demonstrated minimal or no hindlimb movement, those with scores from 8 to 13 (reflecting the intermediate stage of recovery) displayed uncoordinated steps, while rats scoring between 14 and 21 (indicative of the late stage of recovery) exhibited coordinated forelimb and hindlimb movement^57^.

### Histopathological and immunohistochemistry studies

Sixty days following cell transplantation, spinal tissues were harvested for assessing cell engraftment and were stained with anti-β-tubulin III (TUJ-1) and anti-glial fibrillary acidic protein (GFAP) antibodies to identify differentiation into neurons and astrocytes, respectively. At the end of the experiment, the animals were euthanized under deep anesthesia (xylazine 10 mg/kg + ketamine 100 mg/kg i.p.). After induction of deep anesthesia, perfusion with 4% paraformaldehyde in PBS buffer was conducted, and the spinal tissues were then collected and fixed with 4% paraformaldehyde. Subsequently, the tissues underwent processing and were paraffin-embedded (Merck, Germany). Sections of spinal tissues, 5 μm thick, were prepared using a microtome. To assess the number of implanted cells, three sections at intervals of every 20 sections from the lesion epicenter were chosen per rat. After clearance and dehydration of sections, antigen retrieval was performed. The spinal sections were then incubated overnight at 4 °C with human primary antibodies against β-tubulin III and GFAP, following incubation with a blocking solution. Subsequently, the sections were washed with PBS and incubated with goat anti-human secondary antibody for 2 h at room temperature. After PBS washing, nuclei were counterstained with DAPI for 30 min, and the sections were mounted. Labeled stem cells were directly observed using fluorescent microscopy and the OLYSIA Bio Report Soft Imaging System. Cell counting was conducted by identifying dying cells whose nuclei were stained with DAPI. Additionally, Hematoxylin and Eosin (H&E) staining were performed to confirm spinal cord injury.

### Statistical analysis

The findings were presented as mean ± standard deviation (SD). The normality of values was assessed using the Kolmogorov-Smirnov test. A one-way analysis of variance (ANOVA), followed by Tukey’s post hoc test for comparisons, was utilized to statistically analyze BBB scores. Other data were analyzed using independent t-tests. Statistical analysis was conducted using IBM SPSS Statistics for Windows, version 26 (IBM Corp, Armonk, NY, USA), with a significance set at *P* < 0.05.

## Data Availability

The datasets used and/or analyzed during the current study are available from the corresponding author upon reasonable request.

## Abbreviations

DPSCs: Dental pulp stem cells
hDPSCs: Human dental pulp stem cells
MT: Melatonin
IHC: Immunohistochemistry
BBB score: Basso, Beattie, and Bresnahan score
SCI: Spinal cord injury
MSCs: Mesenchymal stem cells
ADSCs: Adipose-derived stem cells
ADMSCs: Adipose-derived mesenchymal stem cells
BMSCs: Bone marrow stem cells
ESC: Embryonic stem cell
iPSC: Induced pluripotent stem cells
DSCs: Dental stem cells
GDNF: Glial cell‑derived neurotrophic factor
BDNF: Brain‑derived neurotrophic factor
CNTF: Ciliary neurotrophic factor
ROS: Reactive oxygen species
ERS: Endoplasmic reticulum stress
PBS: Phosphate-buffered saline
FBS: Fetal bovine serum
MASCIS: Multicenter animal spinal cord injury study
TUJ-1: Beta-III tubulin antibody
GFAP: Glial fibrillary acidic protein
H&E: Hematoxylin and eosin
SD: Standard deviation
ANOVA: Analysis of variance
NeuN: Neuron-specific nuclear protein
HGF: Hepatocyte growth factor
IFN-γ: Interferon gamma

## References

[1] Silva, N. A., Sousa, N., Reis, R. L. & Salgado, A. J. From basics to clinical: a comprehensive review on spinal cord injury. *Prog. Neurobiol.* **114**, 25–57 (2014). 24269804 10.1016/j.pneurobio.2013.11.002

[2] Yuan, S., Shi, Z., Cao, F., Li, J. & Feng, S. Epidemiological features of spinal cord injury in China: a systematic review. *Front. Neurol.* **9**, 683 (2018). 30186222 10.3389/fneur.2018.00683 PMC6113592

[3] Xu, Y. et al. Spinal cord regeneration using dental stem cell-based therapies. *Acta Neurobiol. Exp.* **79**(4), 319–327 (2019). 31885389

[4] Scholpa, N. E. & Schnellmann, R. G. Mitochondrial-based therapeutics for the treatment of spinal cord injury: mitochondrial biogenesis as a potential pharmacological target. *J. Pharmacol. Exp. Ther.* **363**(3), 303–313 (2017). 28935700 10.1124/jpet.117.244806 PMC5676296

[5] Yamamoto, A., Sakai, K., Matsubara, K., Kano, F. & Ueda, M. Multifaceted neuro-regenerative activities of human dental pulp stem cells for functional recovery after spinal cord injury. *Neurosci. Res.* **78**, 16–20 (2014). 24252618 10.1016/j.neures.2013.10.010

[6] Hu, Z.-B. et al. Platelet rich plasma enhanced neuro-regeneration of human dental pulp stem cells in vitro and in rat spinal cord. *Ann. Transl. Med.* **10**(10) (2022). 10.21037/atm-22-1745 PMC9201165 35722381

[7] Dalamagkas, K., Tsintou, M., Seifalian, A. & Seifalian, A. M. Translational regenerative therapies for chronic spinal cord injury. *Int. J. Mol. Sci.* **19**(6), 1776 (2018). 29914060 10.3390/ijms19061776 PMC6032191

[8] Gazdic, M. et al. Stem cells therapy for spinal cord injury. *Int. J. Mol. Sci.* **19**(4), 1039 (2018). 29601528 10.3390/ijms19041039 PMC5979319

[9] Shao, A., Tu, S., Lu, J. & Zhang, J. Crosstalk between stem cell and spinal cord injury: pathophysiology and treatment strategies. *Stem Cell Res. Ther.* **10**(1), 1–13 (2019). 31387621 10.1186/s13287-019-1357-z PMC6683526

[10] Yamazaki, K., Kawabori, M., Seki, T. & Houkin, K. Clinical trials of stem cell treatment for spinal cord injury. *Int. J. Mol. Sci.* **21**(11), 3994 (2020). 32498423 10.3390/ijms21113994 PMC7313002

[11] Gao, L. et al. Progress in stem cell therapy for spinal cord injury. *Stem Cells Int.* (2020). 10.1155/2020/2853650 PMC7661146 33204276

[12] Huang, L., Fu, C., Xiong, F., He, C. & Wei, Q. Stem cell therapy for spinal cord injury. *Cell Transplant.* **30**, 0963689721989266 (2021). 33559479 10.1177/0963689721989266 PMC7876757

[13] Li, Y. et al. Peripheral nerve regeneration using different germ layer-derived adult stem cells in the past decade. *Behav. Neurol.* (2021). 10.1155/2021/5586523 PMC8448597 34539934

[14] Volarevic, V. et al. Ethical and safety issues of stem cell-based therapy. *Int. J. Med. Sci.* **15**(1), 36 (2018). 29333086 10.7150/ijms.21666 PMC5765738

[15] Liau, L. L. et al. Treatment of spinal cord injury with mesenchymal stem cells. *Cell. Biosci.* **10**(1), 1–17 (2020). 32983406 10.1186/s13578-020-00475-3 PMC7510077

[16] Gronthos, S., Mankani, M., Brahim, J., Robey, P. G. & Shi, S. Postnatal human dental pulp stem cells (DPSCs) in vitro and in vivo. *Proc. Natl. Acad. Sci.* **97**(25), 13625–13630 (2000). 11087820 10.1073/pnas.240309797 PMC17626

[17] Karamzadeh, R. & Eslaminejad, M. B. *Dental-Related Stem Cells and Their Potential in Regenerative Medicine* (IntechOpen, 2013).

[18] Ezquer, F. E., Ezquer, M. E., Vicencio, J. M. & Calligaris, S. D. Two complementary strategies to improve cell engraftment in mesenchymal stem cell-based therapy: increasing transplanted cell resistance and increasing tissue receptivity. *Cell Adhes. Migr.* **11**(1), 110–119 (2017). 10.1080/19336918.2016.1197480 PMC5308221 27294313

[19] Liao, N. et al. Antioxidant preconditioning improves therapeutic outcomes of adipose tissue-derived mesenchymal stem cells through enhancing intrahepatic engraftment efficiency in a mouse liver fibrosis model. *Stem Cell Res. Ther.* **11**(1), 1–11 (2020). 32546282 10.1186/s13287-020-01763-y PMC7298967

[20] Zhou, L. N. et al. A comparison of the use of adipose-derived and bone marrow-derived stem cells for peripheral nerve regeneration in vitro and in vivo. *Stem Cell Res. Ther.* **11**(1), 1–16 (2020). 32272974 10.1186/s13287-020-01661-3 PMC7147018

[21] Mendivil-Perez, M. et al. Melatonin enhances neural stem cell differentiation and engraftment by increasing mitochondrial function. *J. Pineal Res.* **63**(2), e12415 (2017). 10.1111/jpi.12415 28423196

[22] Rafat, A., Roushandeh, A. M., Alizadeh, A., Hashemi-Firouzi, N. & Golipoor, Z. Comparison of the melatonin preconditioning efficacy between bone marrow and adipose-derived mesenchymal stem cells. *Cell. J. (Yakhteh)* **20**(4), 450 (2019). 10.22074/cellj.2019.5507 PMC6099139 30123990

[23] Lee, S. J., Jung, Y. H., Oh, S. Y., Yun, S. P. & Han, H. J. Melatonin enhances the human mesenchymal stem cells motility via melatonin receptor 2 coupling with Gαq in skin wound healing. *J. Pineal Res.* **57**(4), 393–407 (2014). 25250716 10.1111/jpi.12179

[24] Chan, Y-H. et al. Melatonin enhances osteogenic differentiation of dental pulp mesenchymal stem cells by regulating MAPK pathways and promotes the efficiency of bone regeneration in calvarial bone defects. *Stem Cell Res. Ther.* **13**(1), 73 (2022). 35183254 10.1186/s13287-022-02744-z PMC8858457

[25] Chang, H. M. et al. Proliferative effects of melatonin on S chwann cells: implication for nerve regeneration following peripheral nerve injury. *J. Pineal Res.* **56**(3), 322–332 (2014). 24499296 10.1111/jpi.12125

[26] Nakade, O., Koyama, H., Ariji, H., Yajima, A. & Kaku, T. Melatonin stimulates proliferation and type I collagen synthesis in human bone cells in vitro. *J. Pineal Res.* **27**(2), 106–110 (1999). 10496146 10.1111/j.1600-079x.1999.tb00603.x

[27] Liao, N. et al. Antioxidant preconditioning improves therapeutic outcomes of adipose tissue-derived mesenchymal stem cells through enhancing intrahepatic engraftment efficiency in a mouse liver fibrosis model. *Stem Cell Res. Ther.* **11**, 1–11 (2020). 32546282 10.1186/s13287-020-01763-y PMC7298967

[28] Ma, Q. et al. Poly (lactide-co-glycolide)-monomethoxy-poly-(polyethylene glycol) nanoparticles loaded with melatonin protect adipose-derived stem cells transplanted in infarcted heart tissue. *Stem Cells* **36**(4), 540–550 (2018). 29327399 10.1002/stem.2777

[29] Karkehabadi, H., Abbasi, R., Najafi, R. & Khoshbin, E. The effects of melatonin on the viability and osteogenic/odontogenic differentiation of human stem cells from the apical papilla. *Mol. Biol. Rep.* **50**(11), 8959–8969 (2023). 37715020 10.1007/s11033-023-08747-0

[30] García-Bernal, D. et al. Melatonin treatment alters biological and immunomodulatory properties of human dental pulp mesenchymal stem cells via augmented transforming growth factor beta secretion. *J. Endod.* **47**(3), 424–435 (2021). 33359532 10.1016/j.joen.2020.12.008

[31] Patil, S. et al. Dose-dependent effects of melatonin on the viability, proliferation, and differentiation of dental pulp stem cells (DPSCs). *J. Person. Med.* **12**(10), 1620 (2022). 10.3390/jpm12101620 PMC9605259 36294759

[32] Asadi-Golshan, R. et al. Efficacy of dental pulp-derived stem cells conditioned medium loaded in collagen hydrogel in spinal cord injury in rats: stereological evidence. *J. Chem. Neuroanat.* **116**, 101978 (2021). 34098013 10.1016/j.jchemneu.2021.101978

[33] Naeimi, A., Zaminy, A., Amini, N., Balabandi, R. & Golipoor, Z. Effects of melatonin-pretreated adipose-derived mesenchymal stem cells (MSC) in an animal model of spinal cord injury. *BMC Neurosci.* **23**(1), 65 (2022). 36384473 10.1186/s12868-022-00752-6 PMC9667651

[34] Mias, C. et al. Mesenchymal stem cells promote matrix metalloproteinase secretion by cardiac fibroblasts and reduce cardiac ventricular fibrosis after myocardial infarction. *Stem Cells* **27**(11), 2734–2743 (2009). 19591227 10.1002/stem.169

[35] Zamini, A. et al. *Osteogenic Differentiation of Rat Mesenchymal Stem Cells from Adipose Tissue in Comparison with Bone Marrow Mesenchymal Stem Cells: Melatonin as a Differentiation Factor* (2008). 18762816

[36] Jamilian, M. et al. Effects of melatonin supplementation on hormonal, inflammatory, genetic, and oxidative stress parameters in women with polycystic ovary syndrome. *Front. Endocrinol.* **10**, 273 (2019). 10.3389/fendo.2019.00273 PMC6527800 31139144

[37] Sakai, K. et al. Human dental pulp-derived stem cells promote locomotor recovery after complete transection of the rat spinal cord by multiple neuro-regenerative mechanisms. *J. Clin. Investig.* **122**(1), 80–90 (2012). 22133879 10.1172/JCI59251 PMC3248299

[38] Darabi, S. et al. Trans-differentiation of human dental pulp stem cells into cholinergic-like neurons via nerve growth factor. *Basic. Clin. Neurosci.* **10**(6), 609 (2019). 32477478 10.32598/bcn.10.6.609 PMC7253808

[39] Darvishi, M. et al. Differentiation of human dental pulp stem cells into functional motor neuron: in vitro and ex vivo study. *Tissue Cell* **72**, 101542 (2021). 33964606 10.1016/j.tice.2021.101542

[40] de Almeida, F. M. et al. Human dental pulp cells: a new source of cell therapy in a mouse model of compressive spinal cord injury. *J. Neurotrauma* **28**(9), 1939–1949 (2011). 21609310 10.1089/neu.2010.1317

[41] Nagashima, K. et al. Priming with FGF2 stimulates human dental pulp cells to promote axonal regeneration and locomotor function recovery after spinal cord injury. *Sci. Rep.* **7**(1), 13500 (2017). 29044129 10.1038/s41598-017-13373-5 PMC5647367

[42] Nicola, F. C. et al. Human dental pulp stem cells transplantation combined with treadmill training in rats after traumatic spinal cord injury. *Braz. J. Med. Biol. Res.* **49**, e5319 (2016). 27509306 10.1590/1414-431X20165319 PMC4988478

[43] Feitosa, M. L. T. et al. Transplantation of human immature dental pulp stem cell in dogs with chronic spinal cord injury. *Acta Cirurg. Bras.* **32**, 540–549 (2017). 28793038 10.1590/s0102-865020170070000005

[44] Song, M., Lee, J-H., Bae, J., Bu, Y. & Kim, E-C. Human dental pulp stem cells are more effective than human bone marrow-derived mesenchymal stem cells in cerebral ischemic injury. *Cell Transplant.* **26**(6), 1001–1016 (2017). 28105979 10.3727/096368916X694391 PMC5657745

[45] Song, M., Jue, S. S., Cho, Y. A. & Kim, E. C. Comparison of the effects of human dental pulp stem cells and human bone marrow-derived mesenchymal stem cells on ischemic human astrocytes in vitro. *J. Neurosci. Res.* **93**(6), 973–983 (2015). 25663284 10.1002/jnr.23569

[46] Bonaventura, G. et al. Dental mesenchymal stem cells and neuro-regeneration: a focus on spinal cord injury. *Cell Tissue Res.* **379**, 421–428 (2020). 31776822 10.1007/s00441-019-03109-4

[47] Demircan, P. C. et al. Immunoregulatory effects of human dental pulp-derived stem cells on T cells: comparison of transwell co-culture and mixed lymphocyte reaction systems. *Cytotherapy* **13**(10), 1205–1220 (2011). 21905956 10.3109/14653249.2011.605351

[48] Ebens, A. et al. Hepatocyte growth factor/scatter factor is an axonal chemoattractant and a neurotrophic factor for spinal motor neurons. *Neuron* **17**(6), 1157–1172 (1996). 8982163 10.1016/s0896-6273(00)80247-0

[49] Hamanoue, M. et al. Neurotrophic effect of hepatocyte growth factor on central nervous system neurons in vitro. *J. Neurosci. Res.* **43**(5), 554–564 (1996). 8833090 10.1002/(SICI)1097-4547(19960301)43:5<554::AID-JNR5>3.0.CO;2-H

[50] Kitamura, K. et al. Hepatocyte growth factor promotes endogenous repair and functional recovery after spinal cord injury. *J. Neurosci. Res.* **85**(11), 2332–2342 (2007). 17549731 10.1002/jnr.21372

[51] Baysal, E., Zırh, E. B., Buber, E., Jakobsen, T. K. & Zeybek, N. D. The effect of melatonin on Hippo signaling pathway in dental pulp stem cells. *Neurochem. Int.* **148**, 105079 (2021). 34048846 10.1016/j.neuint.2021.105079

[52] Mortezaee, K. et al. Melatonin pretreatment enhances the homing of bone marrow-derived mesenchymal stem cells following transplantation in a rat model of liver fibrosis. *Iran. Biomed. J.* **20**(4), 207 (2016). 27130910 10.7508/ibj.2016.04.004 PMC4983675

[53] Tang, Y. et al. Melatonin pretreatment improves the survival and function of transplanted mesenchymal stem cells after focal cerebral ischemia. *Cell Transplant.* **23**(10), 1279–1291 (2014). 23635511 10.3727/096368913x667510

[54] Lee, T-H. Functional effect of mouse embryonic stem cell implantation after spinal cord injury. *J. Exerc. Rehabil.* **9**(2), 230–233 (2013). 24278865 10.12965/jer.130004 PMC3836514

[55] Basso, D. M., Beattie, M. S. & Bresnahan, J. C. A sensitive and reliable locomotor rating scale for open field testing in rats. *J. Neurotrauma* **12**(1), 1–21 (1995). 7783230 10.1089/neu.1995.12.1

[56] Letaif, O. B. et al. Standardization of an experimental model of intradural injection after spinal cord injury in rats. *Clinics* **76**(2021). 10.6061/clinics/2021/e2740 PMC7978664 33787659

[57] Yang, R., Cai, X., Li, J., Liu, F. & Sun, T. Protective effects of MiR-129-5p on acute spinal cord injury rats. *Med. Sci. Monit. Int. Med. J. Exp. Clin. Res.* **25**, 8281 (2019). 10.12659/MSM.916731 PMC6854883 31680116

